# Genetic analysis of the capsule polysaccharide (K antigen) and exopolysaccharide genes in pandemic *Vibrio parahaemolyticus *O3:K6

**DOI:** 10.1186/1471-2180-10-274

**Published:** 2010-11-02

**Authors:** Yuansha Chen, Jianli Dai, J Glenn Morris, Judith A Johnson

**Affiliations:** 1 Department of Pathology, University of Florida, 1600 SW Archer Road, Gainesville, FL 32610, USA; 2 Emerging Pathogens Institute, University of Florida, 2055 Mowry Road, Gainesville, FL 32610, USA

## Abstract

**Background:**

Pandemic *Vibrio parahaemolyticus *has undergone rapid changes in both K- and O-antigens, making detection of outbreaks more difficult. In order to understand these rapid changes, the genetic regions encoding these antigens must be examined. In *Vibrio cholerae *and *Vibrio vulnificus*, both O-antigen and capsular polysaccharides are encoded in a single region on the large chromosome; a similar arrangement in pandemic *V. parahaemolyticus *would help explain the rapid serotype changes. However, previous reports on "capsule" genes are controversial. Therefore, we set out to clarify and characterize these regions in pandemic *V. parahaemolyticus *O3:K6 by gene deletion using a chitin based transformation strategy.

**Results:**

We generated different deletion mutants of putative polysaccharide genes and examined the mutants by immuno-blots with O and K specific antisera. Our results showed that O- and K-antigen genes are separated in *V. parahaemolyticus *O3:K6; the region encoding both O-antigen and capsule biosynthesis in other vibrios, i.e. genes between *gmhD *and *rjg*, determines the K6-antigen but not the O3-antigen in *V. parahaemolyticus*. The previously identified "capsule genes" on the smaller chromosome were related to exopolysaccharide synthesis, not K-antigen.

**Conclusion:**

Understanding of the genetic basis of O- and K-antigens is critical to understanding the rapid changes in these polysaccharides seen in pandemic *V. parahaemolyticus. *This report confirms the genetic location of K-antigen synthesis in *V. parahaemolyticus *O3:K6 allowing us to focus future studies of the evolution of serotypes to this region.

## Background

*V. parahaemolyticus *is a naturally occurring marine bacterium that has been recognized as an important food borne pathogen since a large outbreak occurred in Japan in 1950[1]. Before 1996, no particular serotype of *V. parahaemolyticus *was associated with outbreaks. In that year, there was a major outbreak in Kolkata, India caused by strains with increased virulence and more than half of the patient isolates were serotype O3:K6 [2]. These isolates quickly spread to other countries in Asia, followed by South America, Africa and the United States affecting tens of thousands people and resulting in the first known *V. parahaemolyticus *pandemic [2,3]. Strains from early in the pandemic were all serotype O3:K6 [4,5]. However, the pandemic strains have rapidly evolved to more than 20 serovariants including O3:K6, O4:K68, O1:K25, O1:KUT (K-untypable) and others [2]. The pandemic isolates are closely related (clonal) as shown by pulse-field gel electrophoresis, ribotyping, and multilocus sequence typing (MLST). Therefore, new serotypes seem to have arisen from the original pandemic O3:K6 strain by changes occurring in both the K- (capsule) and the O-antigen. Understanding the mechanism underlying rapid serotype conversion may help us develop improved diagnostics for identifying isolates with pandemic potential.

Eleven O and 65 K serotypes are recognized in *V. parahaemolyticus. *The lipopolysaccharide (LPS) of most Gram-negative bacilli consists of lipid A, core polysaccharide and the highly variable O side chain (O-antigen). The capsular or K-antigen is composed of high molecular weight polysaccharide and forms a dense, high molecular weight coat outside of the bacterial cells. Encapsulated pathogens can become invasive and cause septicemia due to their increased resistance to phagocytosis and complement-mediated killing. K- and O- antigens are generally encoded in discreet loci; but, in limited studies in *V. cholerae *and *V. vulnificus *isolates, O-antigen and K-antigen have been shown to be co-located [6-8]. A third form of polysaccharide, the exopolysaccharide, is a loose slime outside the cell that forms an intercellular matrix in biofilms. In *V. cholerae*, this exopolysaccharide is expressed by cells that display a rugose (wrinkled) colony phenotype [9].

Genetic study of surface polysaccharides in *V. parahaemolyticus *is limited and controversial. Guvener et al. have proposed a locus on chromosome II, VPA1403-VPA1412, for capsular polysaccharide biosynthesis, but have not shown a correlation with the K-antigen [10]. Comparison of restriction fragment polymorphisms of different serotypes led Okura et al to suggest a different locus, around VP214-VP237on chromosome I, for K-antigen genes and a region with homology to the LPS-core polysaccharide region for the O-antigen, but have not experimentally confirmed the function of these regions [11]. To resolve this controversy, we have investigated these putative K-antigen genetic determinants in an epidemic O3:K6 isolate by construction of gene deletions.

## Results

### Polysaccharide gene clusters in *V. parahaemolyticus *O3:K6

**Figure 1:**
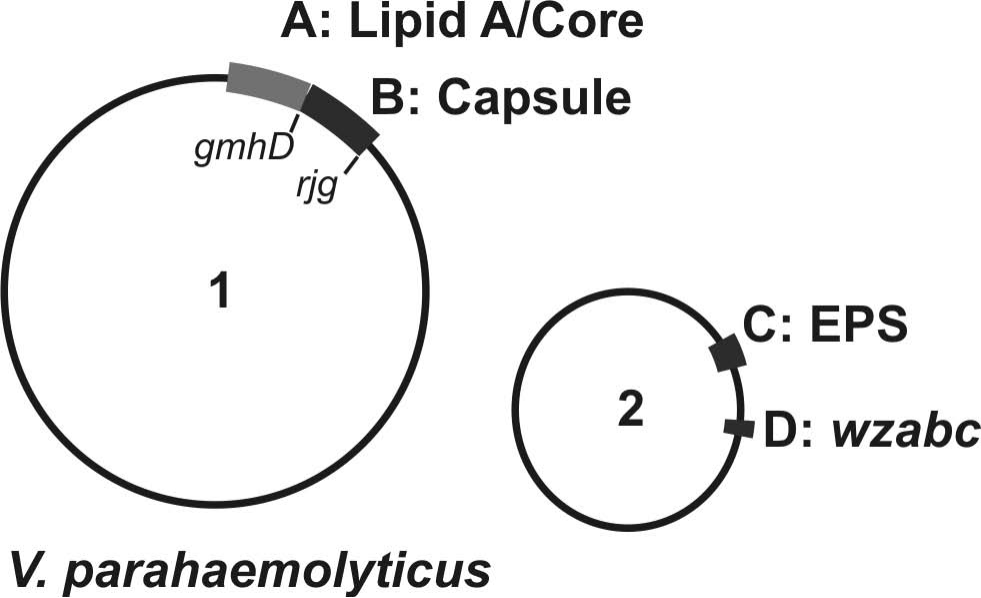
**Gene clusters related to polysaccharide in *Vibrio parahaemolyticus *O3:K6**. Two circles to represent two chromosomes. Function of each region is indicated. A (VP0190-0214), putative lipid A/core region; B (VP0215-0237), K-antigen/capsule region (CPS); C (VPA1403-1412), exopolysaccharide region (EPS); D (VPA1602-1604), putative polysaccharide exportation genes *wza*, *b*, *c*.

From the genome of *V. parahaemolyticus *RIMD2210633, we identified four gene clusters that may relate to surface polysaccharide synthesis judging by their homologs in *V. cholerae *and *V. vulnificus *(Figure 1). Region A includes genes VP0190-0214. Border genes in region A, i.e. VP0190-0191 and VP0211-0214 are homologous to genes in the other species that synthesize lipid A, Kdo or heptoses, which are all signature components of lipid A or core components in LPS. VP0214 is a homolog of *gmhD*, an ADP-L-glycero-D-manoheptose-6-epimerase, which has never been successfully deleted in the other species suggesting that its deletion was possibly lethal. Since there is good homology with known lipid A/core regions and mutations in their genes may be lethal, we have not attempted to delete this region in this study. Region B (VP0215-0237) lies between genes *gmhD *(VP0214) and *rjg *(VP0238), which define the regions for O-antigen biosynthesis in *V. cholerae *serogroups O1, O22, O31, O37 and O139 [7,12-16]. Besides O-antigen, this region also defines the capsule genes for non-O1 *V. cholerae *O31 and O139 [7,13]. In *V. vulnificus*, O-antigen and capsule genes are both located between *gmhD *and *rjg *as well [6]. Previous studies have found similar restriction fragment length polymorphism patterns in region B of strains with the same K serotype suggesting this region may contain the capsule genes [11]. However, region C (VPA1403-VPA1412) in chromosome II was previously identified as the capsule gene region [10]. We deleted genes in region B and C (table 1) to clarify this discrepancy and to answer the question if O- polysaccharide and capsule polysaccharide share the same genes in *V. parahaemolyticus*, as is the case in both *V. cholerae *and *V. vulnificus*.

**Table 1:** *V. parahaemolyticus *strains used in this study

Strain	Description*
VP53	Wild type

∆0220	Deletion of VP0220 (*wbfF*) in region B

∆0220 plus complementation	∆0220 trans-complemented with VP0220

∆CPS	Deletion of VP0219-0237 in region B

∆VP215-218	Deletion of VP0215-0218 in region B

∆EPS	Deletion of VPA1403-1406 in region C

∆EPS plus complementation	∆EPS trans-complemented with VPA1403-1406

∆EPS plus empty vector	∆EPS with pBBR1-MCS2 empty vector

∆*wzabc*	Deletion of *wza*, *wzb *and *wzc *(VPA1602-1604) genes in region D

*All mutants were derived from VP53.

#### Region B determines capsule (K-antigen)

**Figure 2:**
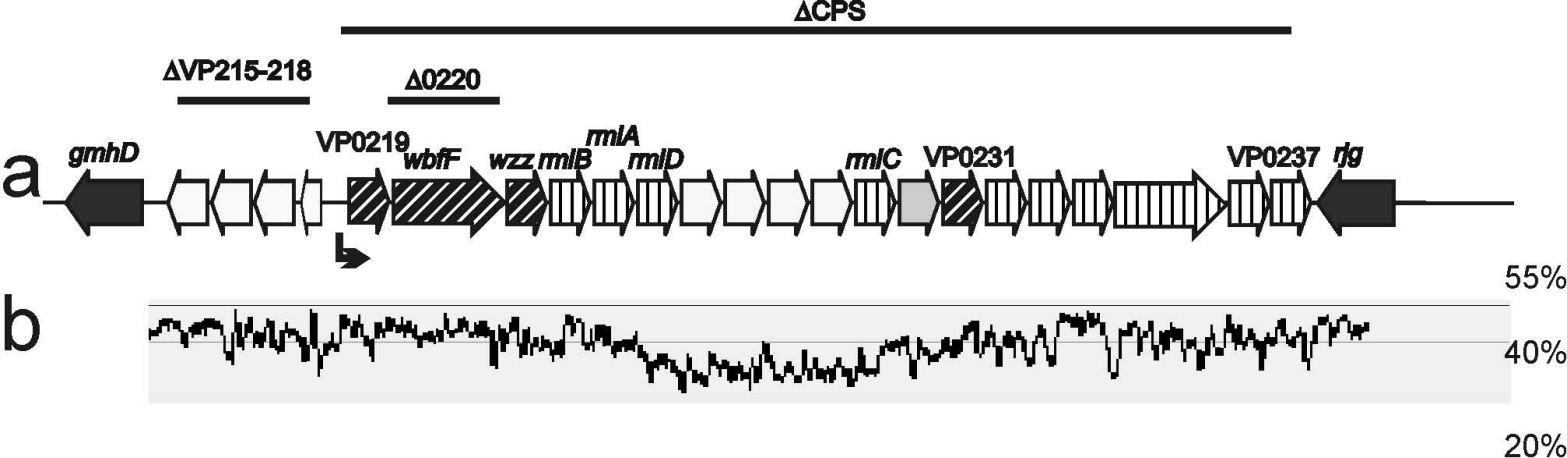
**Capsule (K-antigen) genes in *V. parahaemolyticus *O3:K6**. a) Bars with mutant names above indicate regions deleted in each mutant. Bent arrow indicates promoter. Design patterns of open reading frames indicate different classes of genes: vertical lines, pathway genes; diagonal lines, processing and transportation genes; grey box, glycosyltransferase; white box, functions not clear. b) GC percentage of the sequence in 120 bp windows, aligned to the genes in a.

**Figure 3:**
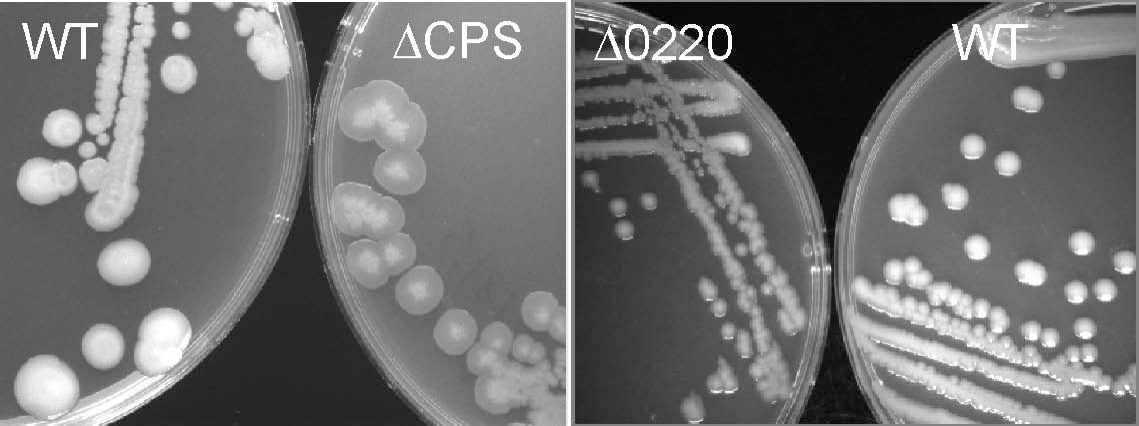
***V. parahaemolyticus *mutants ∆CPS and ∆0220 display translucent phenotype**. Wild type (WT), ∆CPS and ∆0220 have grown on LB agar at 37°C for 24 hours.

According to the annotation in GenBank [17], region B in *V. parahaemolyticus *encodes four hypothetical proteins that are upstream of *gmhD *and transcribed in the same direction, followed by an operon-like structure of 19 open reading frames in the opposite direction (Figure 2, Table 2). To investigate if region B is related to either O-antigen/K-antigen biogenesis in *V. parahaemolyticus*, we deleted the entire 21 kb operon of 19 open frames, VP0219-0237, and replaced it with a Cm cassette (Figure 2). The resulting mutant, ∆CPS, displayed a translucent phenotype consistent with loss of capsule expression, in contrast to an opaque phenotype in the wild type (Figure 3) [18].

**Table 2:** K-antigen/Capsule genes of *V. parahaemolyticus *O3:K6

Gene	S**ymbol**	P**utative function**
VP0214	*gmhD*	ADP-L-glycero-D-manoheptose-6-epimerase

VP0215		hypothetical protein

VP0216		hypothetical protein

VP0217		putative regulator protein

VP0218		hypothetical protein

VP0219		hypothetical protein

VP0220	*wbfF*	capsule assembly protein

VP0221	*wzz*	polysaccharide chain length determinant

VP0222	*rmlB*	dTDP-glucose 4,6 dehydratase

VP0223	*rmlA*	D-glucose-1-phosphate thymidylyltransferase

VP0224	*rmlD*	dTDP-4-dehydrorhamnose reductase

VP0225		hypothetical protein

VP0226		glycosyltranferase

VP0227		hypothetical protein

VP0228		hypothetical protein

VP0229	*rmlC*	dTDP-4-dehydrorhamnose 3,5-epimerase

VP0230		glycosyltranferase

VP0231		UDP-galactose phosphate transferase

VP0232		similar to carbamoyl phosphate synthase

VP0233		hypothetical protein

VP0234		amino transferase

VP0235		putative epimerase

VP0236		UDP-glucose 6-dehydrogenase

VP0237		UTP-glucose-1-phosphate uridylyltransferase

VP0238	*rjg*	hypothetical protein

**Figure 4:**
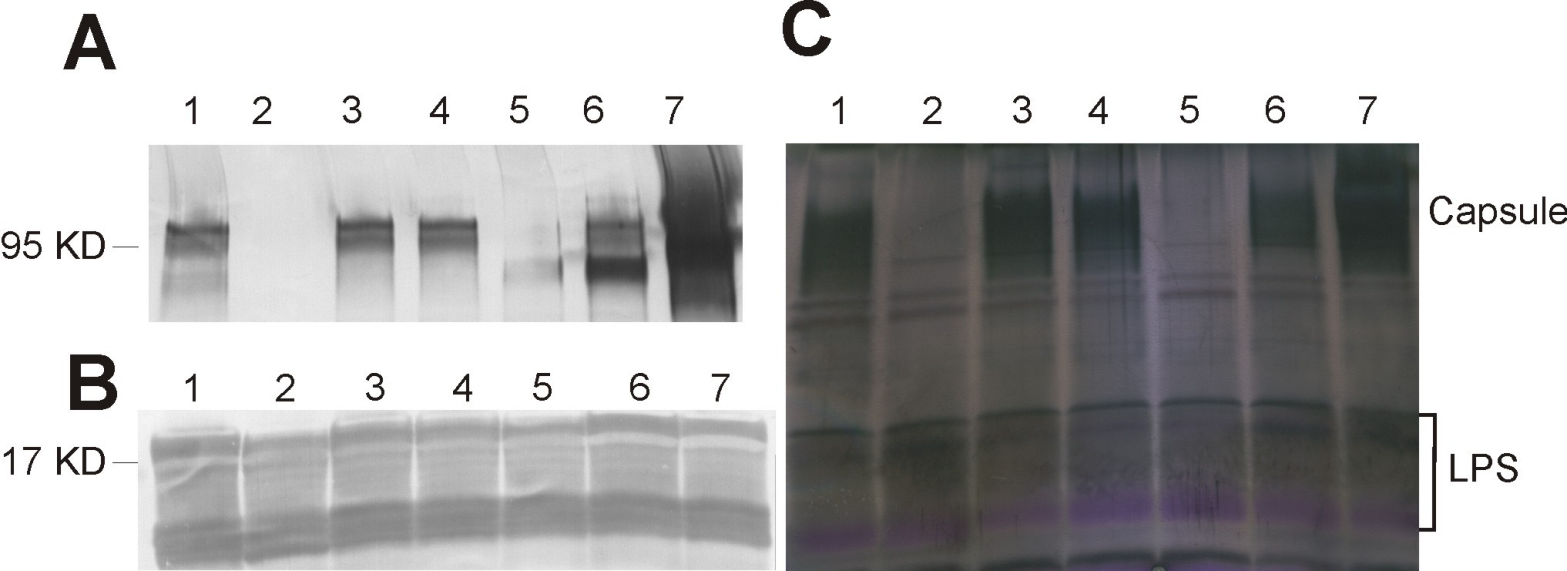
**Immunoblots and stains-all/silver-stain of *V. parahaemolyticus***. Whole cells lysate treated with DNase, RNase and pronase was separated on polyacrylamide gel, transferred to PVDF membrane and probed with K6 specific antiserum (A), or O3 specific antiserum (B). Total polysaccharides were visualized by stains-all/silver-stain on polyacrylamide gel (C). lane 1, wild type VP53; lane 2, ∆CPS mutant; lane 3, ∆EPS mutant; lane 4, ∆*wzabc *mutant; lane 5, ∆0220 mutant; lane 6, ∆0220 mutant with trans-complementation; lane 7, ∆VP215-218 mutant.

We then investigated the immunogenicity of wild type and ∆CPS mutant by immuno-blotting. Whole cell lysate treated with DNase, RNase and pronase was separated on SDS gels, stained with stains-all/silver stain; or blotted to PVDF membrane and probed with O3 or K6 specific antiserum. With the O3:K6 wild type, gels stained with stains-all/silver-stain showed low molecular weight bands circa 17 kDa and high molecular weight bands circa 95 kDa (Figure 4). Immuno-blot developed with O3 antiserum only detected the low molecular weight bands. The low molecular weight is consistent with the LPS/O-antigen. The O3 antiserum bound in the same amount and pattern in ∆CPS mutant as in wild type (Figure 4) indicating that the major operon between *gmhD *and *rjg*, i. e. VP0219-0237, is not involved in O antigen synthesis. Immunoblots developed with K6 antiserum only detected the high molecular weight polysaccharide (Figure 4) in the wild type O3:K6. The high molecular weight of the K-antigen is consistent with capsular polysaccharide. Binding of K6 antiserum was lost in the ∆CPS mutant indicating that region B is required for K antigen biosynthesis. Stains-all/Silver-stain also showed that the high molecular weight capsular polysaccharide was lost in the ΔCPS mutant (Figure 4).

**Figure 5:**
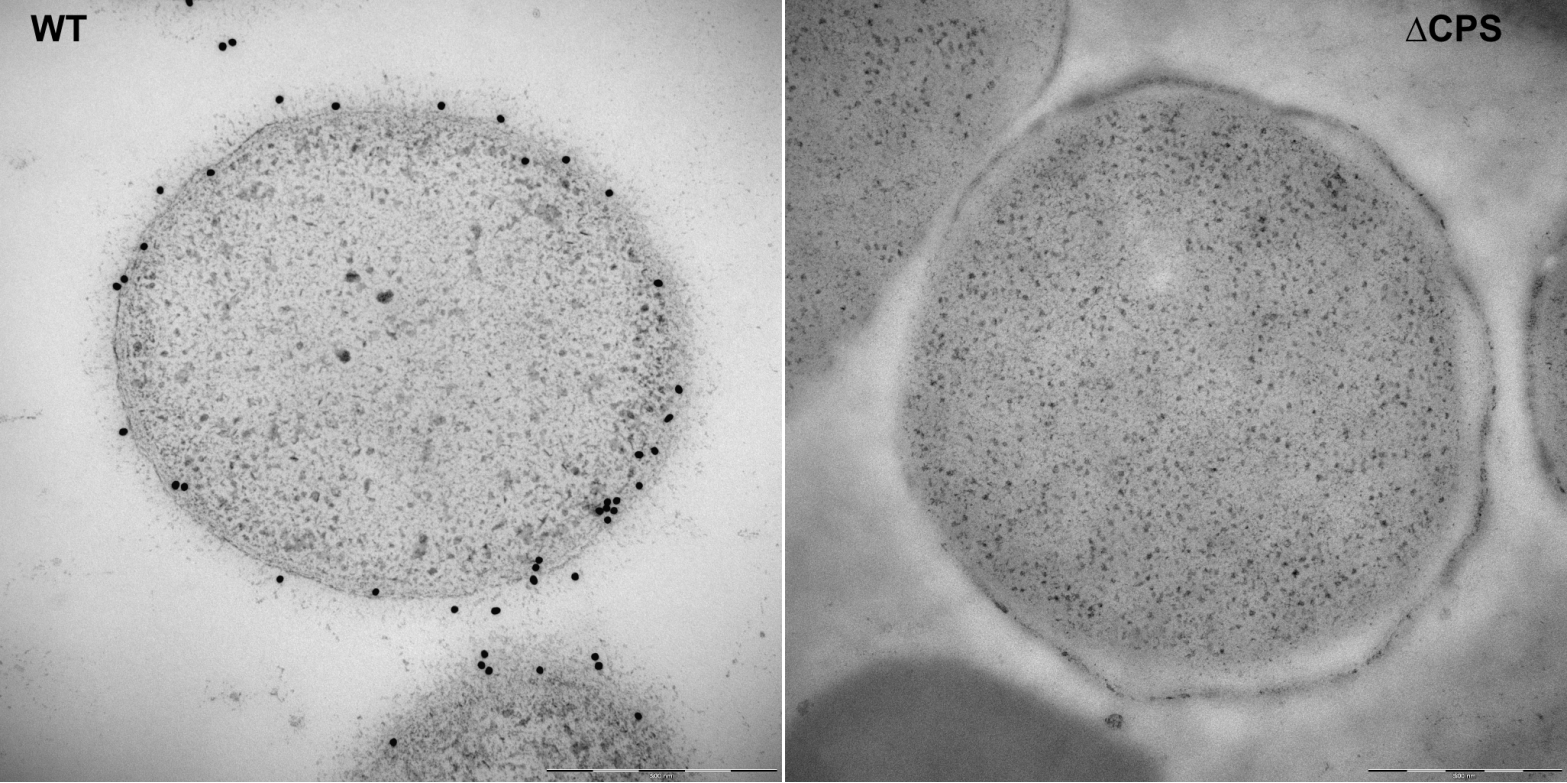
**Immuno-gold labeling TEM of *V. parahaemolyticus *with K6 antiserum**. Thin sections samples were labeled with K6 antiserum, followed by gold attached secondary antibodies. Left, Wild type VP53 (WT), right, ∆CPS mutant. Bar equals to 500 nm.

We further investigated the surface structural change in the ∆CPS mutant by immuno-gold EM using K6 antiserum (Figure 5). The EM image of wild type O3:K6 showed gold particles localized around the exterior of the cell consistent with a capsule-like structure surrounding the cell. This capsule structure was absent from ∆CPS mutant and there was no specific gold particle binding to the cell.

#### K-antigen processing genes

**Figure 6:**
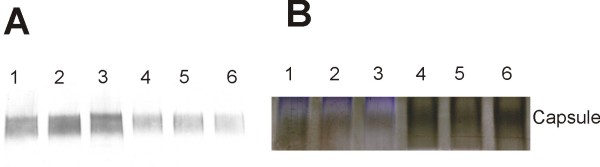
**Immuno blot and stains-all/silver-stain of cell fractions**. Outer membrane (OM) and cytoplasmic (CP) fractions were separated on polyacrylamide gel, then were either transferred to PVDF membrane and probed with K6 specific antiserum (A), or stained with stains-all/silver stain (B). Lane1, wild type CP; lane 2, ∆*wzabc *CP; lane 3, ∆EPS CP; lane 4, wild type OM; lane 5, ∆*wzabc *OM; lane 6, ∆EPS OM.

In order to have some understanding of the capsule/K-antigen biosynthesis pathway, we investigated the polysaccharide processing and assembly genes in the genome of *V. parahaemolyticus*. We identified a small region outside of the K-antigen genes that contains *wza*, *wzb*, and *wzc *genes (Region D, Figure 1). Wza, b and c together constitute an important exportation system in group 1 and group 4 capsules in *E. coli*. A *wza *gene is present in the capsule gene region in both *V. vulnificus *and encapsulated non-O1 *V. cholerae *[7,19]. The *wza *gene in *V. parahaemolyticus *shares 75% and 64% amino acid identity to the *V. vulnificus *and *V. cholerae wza *respectively. To investigate the function of this system in *V. parahaemolyticus *O3:K6, we deleted all three genes in region D from *V. parahaemolyticus *to generate mutant Δ*wzabc*. Δ*wzabc *mutant did not show obvious phenotypic differences to the wild type. Immunoblots and stains-all/silver-stain with whole cell lysate indicated that neither the K antigen nor the O antigen production was affected in the Δ*wzabc *mutant (Figure 4). To further investigate if the capsular polysaccharide accumulated in the cell, as would be anticipated if the exportation of capsule were interrupted, immunoblots and stains-all/silver stain with different cell fractions were performed (Figure 6). There was no difference in K-antigen present outside or inside the cells between the Δ*wzabc *mutant and the wild type. Therefore, our results suggested that the *wza*, *wzb *and *wzc *exportation system was not required by either K6-antigen or O3-antigen production in *V. parahaemolyticus *O3:K6.

However, a K-antigen processing system similar to the O-antigen/capsule polysaccharide genes in *V. cholerae *O139 [13,20,21] is present in *V. parahaemolyticus*. VP0219-0221 are homologous to *wbfE*, *wbfF *and *wzz *genes in *V. cholerae *O139, sharing 49%, 69% and 54% amino acid identities. Therefore a similar capsule processing mechanism may exist for both taxa. We generated an in frame deletion of VP0220, the *wbfF *homolog. Mutant ∆0220 displayed an intermediate level of translucence. Immunoblots indicated that deletion of VP0220 did not affect O3 antigen synthesis (Figure 4). However, the midpoint of the K-antigen band shifted in this mutant, suggesting a role of VP0220 in the later stage of the K-antigen processing. Complementation of ∆0220 with over expressed wild type VP0220 gene restored mostly the pattern of the wild type K antigen (Figure 4). However, there was more reactive material away from the midpoint of the K-antigen band in the complemented mutant than the wild type (Figure 4), possibly due to the over expression of VP0220 or other reasons that remain unclear.

### Other K-antigen region features

A complete set of genes of the rhamnose pathway *rmlBADC *are present in the K-antigen genes of *V. parahaemolyticus*. However, four open reading frames, VP0225-0228, are inserted between the *rmlD *and *rmlC *genes. Analysis of the GC percentage revealed that the average GC percentage in VP0225-0228 is lower than the rest of the genes in this operon (Figure 2). The unusual arrangement of the rhamnose gene order and the mosaic GC percentage pattern indicated that there was a recent recombination event in the K antigen genes.

Between *gmhD *and the K-antigen operon like genes, there are four genes (VP0215-0218) transcribed to the opposite direction (Figure 2). In frame deletion of these four genes led to the over expression of K-antigen polysaccharides (Figure 4), suggesting these genes may have a regulatory role in capsule expression.

#### Region C is the exopolysaccharide gene cluster (EPS)

**Figure 7:**
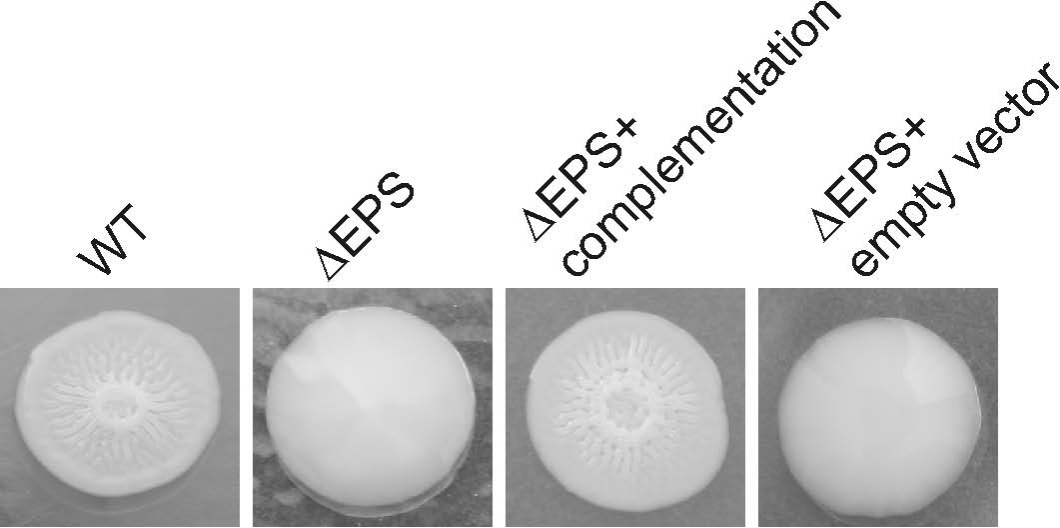
**Colony morphology of *V. parahaemolyticus***. Wild type (WT) *V. parahaemolyticus *displayed rugose phenotype when incubated in APW#3 media followed by 48-72 hours incubation on LB agar. Mutant ∆EPS only displayed smooth phenotype under the same conditions. Complementation of ∆EPS by the EPS genes restored the rugose phenotype while the ∆EPS mutant with empty vector remained smooth.

Region C on chromosome II (VPA1403-1412) contains 10 genes related to polysaccharide biogenesis suggested by Guvener et al to responsible for capsular polysaccharide synthesis in *V. parahaemolyticus *[10]. However, we found that the first 4 genes were similar to exopolysaccharide genes encoding the rugose phenotype in *V. cholerae *[9], sharing the same gene order and 31-54% amino acid identity to their *V. cholerae *homologs. We also compared region C in *V. parahaemolyticus *O3:K6 and O4:K68 (GenBank accession number ACFO00000000) and found that sequences in this region were almost identical in the different serotypes of *V. parahaemolyticus *and thus unlikely to be involved in synthesis of either O- or K-antigen. To clarify the function of this gene cluster, we deleted genesVPA1403-1406 to generate mutant ∆EPS. The ∆EPS mutant displayed an opaque phenotype similar to the wild type on LB agar, and immunoblots showed that neither the K6 nor the O3 antigens were affected in the ∆EPS mutant (Figure 4). Wild type *V. parahaemolyticus *displays phase variation in the colony morphology under certain conditions. Growth in APW#3 media, which induced the rugose phenotype in *V. cholerae *[22], also resulted a rugose colony morphology in *V. parahaemolyticus *with a raised and wrinkled central area (Figure 7). Unlike the wild type, the ∆EPS mutant lost the ability to become rugose after incubation in APW#3 media. Complementation of the ∆EPS mutant by wild type VPA1403-1406 restored the ability to the rugose phase variation (Figure 7). Therefore, we believe that genes in region C, previously referred to as "capsule genes" are not the genes defining the K-antigen, but in fact, are more appropriately designated exopolysaccharide genes.

## Discussion

The genetic region encoding the capsular polysaccharide, or K antigen in *V. parahaemolyticus *has been controversial, with two different investigators suggesting different loci [10,11]. In our study, construction of gene deletions with confirmation of loss of binding K6-specific antiserum in immunoblots provided solid evidence that the region between genes *gmhD *and *rjg *(VP0215-0237) on chromosome I was the genetic determinant of the K6-antigen in the pandemic *V. parahaemolyticus *O3:K6 serotype. This antigen consists of high molecular weight polysaccharide that is located on the surface of the cell. Loss of this antigen resulted in a translucent colony morphology. These data are consistent with the K6 antigen being a typical vibrio capsular polysaccharide. Our study supports the location suggested by Okura et al as encoding the K-antigen [11]. Although O-antigen genes have been identified in a conserved locus between genes *gmhD *and *rjg *in both *V. cholerae *and *V. vulnificus*, our study found that this locus in *V. parahaemolyticus *was not involved in O-antigen biosynthesis. We also showed that gene cluster referred to as "capsule" genes by Guvener et al (VPA1403-VPA1412) was not related to either K-antigen capsule polysaccharide or O-antigen but was instead related to exopolysaccharide production, which causes rugose phase variation. We suggest reserving the term "capsule" for K-antigen polysaccharides and referring to the rugose related polysaccharide exopolysaccharide.

Our understanding of the major surface polysaccharides in *V. parahaemolyticus *had been limited, in part, due to our limited ability to perform genetic manipulations in this species. Genetic manipulation of genes in *V. parahaemolyticus *was previously achieved by first cloning the DNA of interest into a suicide plasmid that cannot replicate in *V. parahaemolyticus*, propagating the plasmid in an *E. coli *host, then transferring the plasmid from *E. coli *to *V. parahaemolyticus *by conjugation, followed by counter selection against the *E. coli *host and screening for mutants of *V. parahaemolyticus *[23]. The procedure is tedious and time consuming. There are few reports using electroporation in *V. parahaemolyticus *and no report of successful chemical transformation [24,25]. We tested electroporation on *V. parahaemolyticus *and had limited success with plasmid DNA but no success with linear DNA (data not shown). Chemical transformation was also not successful. Therefore we sought alternative methods for targeted gene deletion in *V. parahaemolyticus*. Meibom et al. reported that *V. cholerae *became competent and took up foreign DNA when cultured with chitin [26]. The chitin based transformation method was later successfully adapted for *V. vulnificus *[27]. We modified the chitin based transformation technique and developed a rapid method to mutate genes in *V. parahaemolyticus*. On average, 150 mutants were obtained from each transformation. Since only one mutant is needed in most cases, this transformation efficiency will satisfy most deletion applications in *V. parahaemolyticus*.

Capsule biogenesis in *E. coli *is classified into 4 groups. Exportation of group 1 and 4 capsules rely on Wza proteins, while group 2 and 3 may rely on CPSM and CPST proteins [28]. Previous research has shown that capsules in *V. cholerae *O31 and *V. vulnificus *have similarities to *E. coli *group 1- or group 4 capsules; with a *wza *gene inside the capsule gene cluster [6,7,19]. Genomic analysis also revealed that a *wza *gene was present in the putative capsule regions in the other published genomes of *V. vulnificus *and non-O1, non-O139 *V. cholerae *[29]. In contrast, the *wza *gene was present in *V. parahaemolyticus*, but was not within the capsular polysaccharide region. Furthermore, mutagenesis of this gene showed it was not required for K antigen biosynthesis. Deletion of *wbfF *changed the pattern of the K-antigen seen on immunoblots, suggesting that this gene may play a role in assembly of the capsule. However, the processing of K-antigen by the *wbfF *gene and possibly the adjacent *wzz *gene, and the regulation role of the upstream genes will require further investigation.

In both *V. cholerae *and *V. vulnificus *the capsule and O-antigen genes lie in a region similar to the O-antigen region of enteric, such as *E. coli*, and that specific genes may be shared by both biosynthetic pathways [6,7]. Pandemic *V. parahaemolyticus *has changed rapidly in both O and K types, leading to the hypothesis that the genetic determinants of O and K also share the same genetic locus thus allowing a single genetic event to alter the structure of both antigens. However, our finding is not consistent with this hypothesis. Our experiments clearly demonstrated that genes determining the K-antigen in pandemic *V. parahaemolyticus *were located in the region determining both surface polysaccharides in the other vibrios, but that the O-antigen genes are located elsewhere. From our data and Okura et al's observations on polysaccharide genes, we speculate that the region with homology to LPS core regions may be playing the role of O antigen. This speculation is consistent with the finding that the LPS in *V. parahaemolyticus *are rough type [30]. Since the core genes are adjacent to the capsule genes, they could still be replaced in the same recombination event and give rise to both new O- and K-antigens. Analysis of putative O and K antigen genes in a different serotype O4:K68 revealed that these regions are distinct from those of O3:K6 serotype despite their highly similar genetic backbones [11] and suggested both the O and K regions were replaced during the serotype conversion (Chen et al: Comparative genomic analysis of *Vibrio parahaemolyticus*: serotype conversion and virulence, submitted).

## Conclusion

Understanding of the genetic basis of O- and K-antigens is critical to understanding the rapid changes in these polysaccharides seen in pandemic *V. parahaemolyticus. *This is also important in understanding the virulence of *V. parahaemolyticus *as the O- and K-antigens represent major surface antigens responsible for protective immunity. In this study, we found the O and K genes were separated in *V. parahaemolyticus *but their locus maybe adjacent. This report also confirms the genetic location of K-antigen synthesis in *V. parahaemolyticus *O3:K6 allowing us to focus future studies of the evolution of serotypes to this region.

## Methods

### Bacterial strains and growth condition

At the time of this study, we didn't have access to the sequenced strain RIMD 2210633 and numerous studies showed that the pandemic strains of *V. parahaemolyticus *O3:K6 are highly clonal and homogenous in their genomes. In particular, the polysaccharide genes are almost identical in RIMD2210633 and two O3:K6 pandemic strains sequenced (Chen et al: Comparative genomic analysis of *Vibrio parahaemolyticus*: serotype conversion and virulence, submitted), and primers based on RIMD2210633 sequence successfully amplified target DNA from VP53, an O3:K6 pandemic strain isolated from a patient in Kolkata, India in 1996 [5], and subsequent sequence was confirmed to be identical to RIMD2210633. Thus all mutants were generated from *V. parahaemolyticus *VP53. Unless otherwise stated, bacteria were cultured in LB broth or LB agar at 37°C. Antibiotics were added in the following concentration when needed: chloramphenicol at 10 μg/ml, and Kanamycin at 50 μg/ml for *Escherichia coli *and 100 μg/ml for *V. parahaemolyticus*.

To induce rugose phenotype, a single colony was inoculated into 2 ml APW#3 broth [22], incubated at 37°C statically for 48 hours. Then 1 μl of culture was spotted on LB agar plate and incubated at 30°C for 48-72 hours. Pictures were taken when colony size reached about half centimeter.

### Construction of Mutants

Genetic regions to be targeted and primer sequences were determined based on the annotation of *V. parahaemolyticus *genome RIMD2210633 (GenBank Accession BA000031 and BA000032). Several mutants, including a mutation deleting the entire K-antigen structural gene operon on chromosome I (VP0219-0237), several partial deletion mutations in the region on chromosome I (VP0215-0218 and VP0220 gene), and a deletion mutation of exopolysaccharide region in chromosome II (VPA1403-1406) as well as a deletion mutation in a separate region containing polysaccharide transport genes *wza*, *wzb*, and *wzc *were constructed (Table 1). Polymerase Chain Reaction (PCR) was performed using Taq DNA polymerase (Thermo Fisher, Waltham, MA). PCR products were purified on Qiagen PCR purification columns (Qiagen, Valencia, CA). Restriction enzymes were purchased from New England Biolabs (Ipswich, MA).

DNA was prepared for crossover recombination by overlapping PCR. First, three DNA fragments were amplified by PCR separately, including a fragment (500-1000 bp) upstream of targeted gene in *V. parahaemolyticus*, a fragment (500-1000 bp) downstream of targeted gene in *V. parahaemolyticus *and a chloramphenicol resistant gene (Cm) in pKD3 [31]. The 3' end of the reverse primer in the upstream DNA was complementary to the forward primer of Cm, and the 5' end of the forward primer of downstream DNA was complementary to the reverse primer of Cm. Then the three fragments were mixed and assembled into one piece in a second PCR reaction where the product was amplified by primers at the two extremes. Genes deleted and primers used are listed in (Table 3). Two to four micrograms of PCR product were used to transform *V. parahaemolyticus *VP53.

**Table 3:** Primers used in this study

Target	Location	Primer sequence
Cm cassette	pKD3 forward 1	GTGTAGGCTGGAGCTGCTTC
	pKD3 reverse 1	CATATGAATATCCTCCTTA
	pKD3 forward 2	ACCTGTGACGGAAGATCACTTCG
	pKD3 reverse 2	AGGAACTTCATTTAAATGGCGCG

flanking sequence of VP0220 (*wbfF*) gene	upstream forward*	CCCAGCCATAACTAACACTAACCCGT
	upstream reverse	GAAGCAGCTCCAGCCTACACGATAATTCGCTATTTAAATCGAGAGTTAAA
	downstream forward	TAAGGAGGATATTCATATGGGAACGACAAGATCATTCCAATCAG
	downstream reverse*	TAGGCTAAGTTCTGAGAGGTTTCCG

flanking sequence of CPS region VP0219-0237	upstream forward	AATACTAGTGAGCTGTGTTCTTCATTATTAATCCT
	upstream reverse	CGAAGTGATCTTCCGTCACAGGTGCAGTGAATGTCTGTTAACTCT
	downstream forward	CGCGCCATTTAAATGAAGTTCCTCAGGCTCGTTACCAATGTGCT
	downstream reverse	GCCAATTATCCTAGACTCACCACT

flanking sequence of VP0215-0218	upstream forward	CACCAGCATTGATCTGGTTATTCA
	upstream reverse	CGCGCCATTTAAATGAAGTTCCTTATTTAAGGGAGCTTCGGCTCCCT
	downstream forward	CGAAGTGATCTTCCGTCACAGGTAGAGCTGTGTTCTTCATTATTAATCC
	downstream reverse	TCGGCATTAGAGTAGCTCACTAACG

flanking sequence of EPS genes VPA1403-1406	upstream forward ^$^	CCACTACCCACAGAACCGCTTTGT
	upstream reverse	CGAAGTGATCTTCCGTCACAGGTATGACCTAGTTTCCCTTCTAGCA
	downstream forward	CGCGCCATTTAAATGAAGTTCCTAGCCAGGTTTAACCAACATATTGA
	downstream reverse^$^	ACCACTCCAAAGGGTAGTGGTGA

flanking sequence of *wza*, *wzb*, *wzc *genes	upstream forward	CAGGGAATCAAGCATACGTTGAA
	upstream reverse	CGAAGTGATCTTCCGTCACAGGTATTCATCTGACGTAAAGAGCGA
	downstream forward	CGCGCCATTTAAATGAAGTTCCTATCTAGATCGCTAATTTGACCAAA
	downstream reverse	GAGCAGCAAAGCTGCAGATTGA

*Primers to amplify DNA for ∆0220 complementation.

^$^Primers to amplify DNA for ∆EPS complementation.

### Chitin based transformation

Transformations were performed using a modification of the chitin based method of Meibom [26], which has been successfully used in *V. vulnificus *[27]. *V. parahaemolyticus *was cultured to an optical density of 0.4 - 0.6, pelleted and resuspended to an optical density of 0.2 in sterile seawater. Two ml of this culture were added to pieces of sterile crab shell in a culture plate and incubated at 30°C overnight. The next day, culture supernatant was discarded and 2 ml of fresh sea water, along with 2-4 μg of DNA that was prepared as described above, were added. The mixture was returned to 30°C and incubated overnight. Bacteria were then released from crab shells by vortexing and plated on chloramphenicol agar. Deletions in mutants were confirmed by PCR and sequencing.

### Complementation of mutants

DNA fragments deleted in the ∆0220 or ∆EPS mutants were amplified from the wild type genomic DNA (primers listed in Table 3) and cloned into the BamHI and XmaI site of plasmid pBBR1-MCS2 [32]. The resulting plasmids were propagated in *E. coli *S17pir and then mobilized d into *V. parahaemolyticus *mutants by conjugation as described previously[7]. All constructs were confirmed by restriction enzyme digestion patterns and sequencing.

### Immunoblots

To prepare whole cell lysate for polysaccharide analysis, one ml of bacteria culture grown to an O.D. ~1.0 was pelleted, resuspended in 100 μl cell lysis solutions (200 μg/ml lysozyme, 50 μg/ml DNase, 100 μg/ml RNase A) per O.D. and incubated at 37°C for one hour. SDS was added to the solution to 0.5% final concentration and incubated for an additional 30 min. Pronase was then added to 100 μg/ml and samples were incubated at 37°C overnight. Samples were mixed with equal amount of sample buffer (Biorad), boiled for 10 min, separated in a 15% SDS polyacrylamide gel and then transferred to PVDF membranes (Bio-Rad, Hercules, CA). Cell fractions were prepared as described by Koga and Kawata [33]. Briefly, bacteria were treated with lysis buffer (0.6 M sucrose, 100 μg/ml lysozyme, 2.5 mM EDTA and 50 mM Tris-HCl, pH 8.0) at 37°C for 20 min, and then centrifuged at 8000 g for 15 min. The supernatant represented the outer membrane fraction and the pellet represented the cytoplasmic fraction. Cell fraction samples were then treated with DNase and RNase followed by pronase. Aliquots equal to 1 × 10^8 ^cells were separated and blotted as described above. The membranes were blocked with 3% skim milk, and incubated with O3 or K6 specific typing sera (Denka Seiken, Japan), followed by binding with a secondary goat anti-rabbit antibody conjugated with alkaline phosphatase (Bio-Rad). Alkaline phosphatase activity was detected by GAR-AP detection kit (Bio-Rad).

### Stains-all/silver-stain

Polysaccharides were stained by a combination of stains-all/silver-stain method adapted from [34]. After electrophoresis, polyacrylamide gel was fixed following the fixative step as instructed by the silver stain plus kit (Biorad). The gel was then washed with water four times, 10 min each, to ensure the removal of SDS. The gel was stained for 2 hours with a solution containing 4 mg/ml stains-all (MP Biomedicals), 5% formamide, 25% isopropanol and 15 mM Tris-HCL, pH8.8. The gel was de-stained with water until background became clear (about 30 min). Silver stain was then performed following the staining and developing step as instructed by the silver stain plus kit.

### Immuno-gold EM

Immuno-gold EM was performed in the Interdisciplinary Center for Biotechnology Research at the University of Florida. *V. parahaemolyticus *samples were treated by high-pressure freezing, followed by freeze-substitution, embedded in EPOXY resin and thin sectioned. Samples were then labeled with K6 antiserum, followed by gold-labeled secondary antibodies.

## Authors' contributions

YC, JGM and JAJ conceived the study. YC and JD designed and performed the experimental works. YC and JAJ drafted the manuscript. All authors read and proved the final manuscript.
